# Single-Frequency Ultrasound-Based Respiration Rate Estimation with Smartphones

**DOI:** 10.1155/2018/3675974

**Published:** 2018-05-06

**Authors:** Linfei Ge, Jin Zhang, Jing Wei

**Affiliations:** ^1^Department of Computer Science and Engineering, Southern University of Science and Technology, Shenzhen, Guangdong, China; ^2^Department of Electrical and Electronic Engineering, Southern University of Science and Technology, Shenzhen, Guangdong, China

## Abstract

Respiration monitoring is helpful in disease prevention and diagnosis. Traditional respiration monitoring requires users to wear devices on their bodies, which is inconvenient for them. In this paper, we aim to design a noncontact respiration rate detection system utilizing off-the-shelf smartphones. We utilize the single-frequency ultrasound as the media to detect the respiration activity. By analyzing the ultrasound signals received by the built-in microphone sensor in a smartphone, our system can derive the respiration rate of the user. The advantage of our method is that the transmitted signal is easy to generate and the signal analysis is simple, which has lower power consumption and thus is suitable for long-term monitoring in daily life. The experimental result shows that our system can achieve accurate respiration rate estimation under various scenarios.

## 1. Introduction

Respiration is one of the most important vital signs for human beings. Many chronic diseases can be detected through respiration monitoring, such as asthma and chronic obstructive pulmonary diseases [1, 2]. Respiration monitoring can also reflect the users' sleep and emotion status. Respiration rate detection is a key function in respiration monitoring [3]. Most existing respiration rate detection devices are wearable, for example, respiration belts [4] and oronasal airflow meters [5]. Users need to wear these devices on their chests or faces during respiration monitoring, which is extremely inconvenient. Therefore, these devices are mainly applicable for medical examination for disease diagnosis, but they are not suitable for long-term everyday monitoring.

To overcome the disadvantage of the wearable devices, recently, researchers started investigating noncontact respiration monitoring methods. Some works use wireless signals to detect the respiration rate. For example, Abdelnasser et al. leveraged the WiFi signals [6–10], Lazaro et al. used the UWB signals [11, 12], and Rahman et al. used a microwave radar to detect respiration [13–15]. These systems require deploying extra wireless transceivers to transmit and receive wireless signals, which makes the system expensive. Instead of specially deployed wireless transceivers, some researchers proposed using smartphones to detect vital signs, which is easier to access in daily life. Some works used built-in inertial sensors in smartphones to monitor vital signs [16–19], while others leveraged ultrasonic signals to conduct respiration detection and sleep monitoring [20, 21]; they utilized built-in speakers and microphones in smartphones to play and record ultrasound signals and extract useful information such as respiration patterns from them [22]. In our paper, we will also use ultrasound as the media to detect the respiration rate. Actually, ultrasound is a proper medium for vital sign detection, as it can be easily generated by smartphones, which are taken along by people all the time and are suitable for long-term monitoring. Besides, ultrasound is a mechanical wave; therefore, users do not need to worry about the electromagnetic radiation for long-term monitoring. Because of the above advantages, ultrasound is also considered to be used for other applications, such as activity recognition and human computer interaction [23–25]. However, in existing ultrasound-based monitoring systems, complicated signals such as frequency modulated continuous wave (FMCW) or orthogonal frequency division multiplexing (OFDM) are used as the carrier to conduct object detection, which requires complicated modulation and demodulation signal processing modules. The complexity in signal generation and signal processing leads to large power consumption, which is not suitable for power-limited mobile devices such as smartphones.

In this paper, to overcome the above-mentioned limitation of existing solutions, we propose a smartphone-based respiration rate detection system using a single-frequency ultrasonic signal, which enables simple signal processing and low power consumption. We observed that when a single-frequency cosine ultrasonic signal is transmitted by the speaker and the signals reflected by the human being are collected using the built-in microphone, the signal strength of the received signal can reflect the breath pattern of the person being detected. The reason is that the received signal is a combination of the reflected signal and the line-of-sight signal. The chest movement of the respiration activity changes the distance between the speaker and the microphone and, therefore, changes the phase of the reflected signal, which finally results in the amplitude of the total received signal changing periodically. By analyzing the amplitude of the received signal, the respiration rate can be correctly detected. In our proposed system, to detect the respiration rate, we only need to calculate the amplitude of the signal, without complicated frequency domain analysis. Our method guarantees a high detection accuracy while keeping the analysis relatively simple.

The contributions of our paper are as follows. First, we make an observation that the received signal strength of the single-frequency ultrasound signal can reflect the respiration pattern of the user, by theoretical analysis. Second, we propose a smartphone-based respiration rate detection system utilizing single-frequency ultrasonic signals. We also design a rate detection algorithm to estimate the respiration rate based on the amplitude of the received signal. Third, we implement the system on an Android smartphone and the experimental result shows that our system can achieve accurate respiration rate estimation results under various testing scenarios.

## 2. Ultrasonic Signal Analysis

In this section, we will analyze the characteristics of the received ultrasonic signal when a single-frequency signal is transmitted by the speaker and reflected by the tester. The analysis result shows that the strength of the received signal reflects the respiration rate; thus, by detecting the signal strength, the respiration rate can be estimated.

### 2.1. Overview

In this paper, we use the built-in speaker of a smartphone to generate single-frequency ultrasound of 20 kHz. Most off-the-shelf smartphones can generate sound up to 22 kHz using their built-in speakers [20, 21]. The smartphone is placed in front of the tester. The ultrasound signal is reflected by the human body and captured by the built-in microphone of the smartphone. The received signal is mainly composed of two parts. One is the signal directly propagated from the speaker to the microphone. The other is the part that is reflected by the user's moving chest. These two signals have a superposition at the receiving end. Because of the movements of the chest while breathing, the received signal varies. In the following subsection, we will derive the received signal strength of the composed signal. We observed that the signal strength varies along with the respiration. From the amplitude of the received signal, we can extract the respiration waveform and estimate the respiration rate.

### 2.2. Receiving Signal Analysis

In our system, we use the speaker and microphone of a smartphone to transmit and receive ultrasonic signals. At the transmitter end, the speaker emits a single-frequency cosine signal

(1) $$\begin{array}{c} S\left(\;t\;\right)=A\cos\left(\;2\pi f_{s}t\;\right), \end{array}$$

where *A* is the amplitude and *f*_*s*_ is the frequency of the generated ultrasound signal.

The received signal is a superposition of two components: the static signal which propagates directly from the speaker to the microphone and is reflected by the static reflectors and the dynamic one which is caused by the movements of the tester's chest.

For the static component, it contains the line-of-sight signal directly transmitted from the speaker to the microphone and the signals reflected by static objects around. The static reflectors only change the phase of the signal without changing its frequency; therefore, the static component, which is the sum of all static rays, can be written as follows:

(2) $$\begin{array}{c} R_{s}\left(\;t\;\right)=A_{1}\cos\left(\;2\pi f_{s}t+\phi_{s}\;\right), \end{array}$$

where *A*_1_, *f*_*s*_ are the amplitude and frequency of the sound signal, respectively, and *ϕ*_*s*_ is a constant phase change.

For the dynamic component, the periodical movement of the chest causes a periodical distance change between the smartphone and the chest. Thus, the propagation delay varies because of the chest motion. Therefore, the phase also changes periodically. So, the dynamic component can be written as follows:

(3) $$\begin{array}{c} R_{d}\left(\;t\;\right)=A_{2}\cos\left(\;2\pi f_{s}t+\phi_{d}\left(\;t\;\right)\;\right), \end{array}$$

where *A*_2_, *f*_*s*_ are the amplitude and frequency of the sound signal. *ϕ*_*d*_(*t*) is the periodical phase change caused by the chest movement, which is written as

(4) $$\begin{array}{c} \phi_{d}\left(\;t\;\right)=\frac{2\pi f_{s}\left(D_{0}+D\cos\left(2\pi f_{b}t\right)\right)}{\nu }, \end{array}$$

where *D*_0_ is the constant distance of the propagation path, *D* and *f*_*b*_ are the amplitude and frequency of the chest movement while breathing, and *ν* is the speed of sound. Here, *D*_0_ + *D*cos⁡(2*πf*_*b*_*t*) is the distance between the smartphone and the chest while breathing, and (*D*_0_ + *D*cos⁡(2*πf*_*b*_*t*))/*ν* is the propagation delay.

At the receiver end, the received signal is the superposition of the static component and dynamic component:

(5) $$\begin{array}{c} R\left(\;t\;\right)=R_{s}\left(\;t\;\right)+R_{d}\left(\;t\;\right)=A_{r}\left(\;t\;\right)\cos\left(\;2\pi f_{s}t+\varphi \;\right), \end{array}$$

where

(6) Art=A12+A22+2A1A2cos⁡ϕs−ϕdt,

(7) tan⁡φ=A1cos⁡ϕs+A2cos⁡ϕdtA1sin⁡ϕs+A2sin⁡ϕdt.

Bringing (4) into (6), we have

(8) Art=A12+A22+2A1A2cos⁡ϕs−2πfsD0+Dcos⁡2πfbtν=A12+A22+2A1A2cos⁡ϕs−2πfsD0ν−2πfsDνcos⁡2πfbt.

In the above expression of *A*_*r*_(*t*), cos⁡(2*πf*_*b*_*t*) varies with *t* at a frequency of *f*_*b*_, which is the breathing rate. So, the amplitude of the received signal *A*_*r*_(*t*) varies periodically at the same frequency with breath. Therefore, the signal strength, which is the square of the amplitude, follows the same changing pattern. Therefore, by detecting the received signal strength, we can estimate the chest movement of a person.

## 3. Respiration Rate Estimation Algorithm

**Figure 1:**
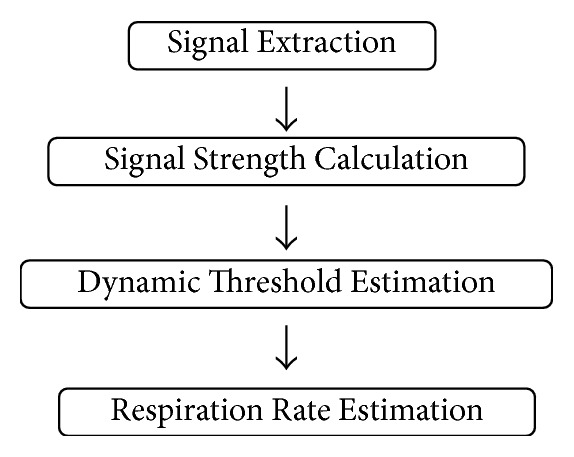
Algorithm overview.

In this section, we present our respiration rate estimation algorithm based on single-frequency ultrasound signals. We aim to run our algorithm and make it work well on smartphones. Considering the limited resources and power supply of smartphones, we try our best to reduce the complexity of our algorithm. We are trying to find the most effective way instead of the most accurate way for signal processing. Our system is composed of four stages: signal extraction, signal strength calculation, dynamic threshold estimation, and respiration rate estimation, as shown in Figure 1.

In the first stage, the smartphone generates an inaudible 20 kHz ultrasound signal, plays it with the built-in speaker, and records the signal using the microphone. The recording process is to sample the received sound signal *R*(*t*) with sampling rate *F*_*s*_, which achieves a discrete signal:

(9) $$\begin{array}{c} X_{n}=R\left(\;\frac{n}{F_{s}}\;\right),\;n=1,2,3,\ldots . \end{array}$$

In the second stage, we calculate the received signal strength. From the analysis in Section 2, we know that the received signal strength changes at the same frequency as the breath. We can estimate the respiration rate based on the received signal strength *P*_*m*_. Although the audio file is sampled with a high sampling frequency *F*_*s*_, the received signal strength *P*_*m*_ can be calculated at relatively low frequency *F*_*p*_, where *F*_*p*_ = *F*_*s*_/*K* and *K* is the coefficient that is used to reduce the sampling rate of signal strength. The signal strength *P*_*m*_ can be defined as the average of the signal strength of *K* samples in *X*_*n*_:

(10) $$\begin{array}{c} P_{m}=\frac{1}{K}\overset{m*K}{\underset{n=\left(m-1\right)*K+1}{\sum }}X_{n}^{2},\;m=1,2,3,\ldots . \end{array}$$

Then, we smooth the received signal strength using a moving average filter with a window size of *W* points. The smoothed signal strength ${\bar{P}}_{m}$ is given by

(11) $$\begin{array}{c} {\bar{P}}_{m}=\frac{1}{W}\overset{m}{\underset{i=\max\left\{i-W,1\right\}}{\sum }}P_{i}\;\left(m=1,2,3,\ldots \right). \end{array}$$

**Figure 2:**
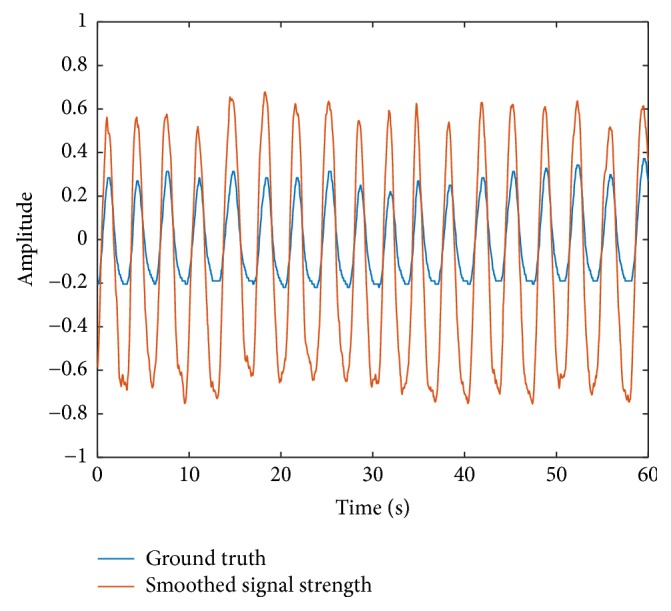
Smoothed signal strength compared with ground truth.

Figure 2 shows the smoothed received signal strength ${\bar{P}}_{m}$ as well as the ground truth achieved by respiration belt SCHUHFRIED Biofeedback Xpert [26]. The result shows that the smoothed signal strength matches the ground truth well.

**Figure 3:**
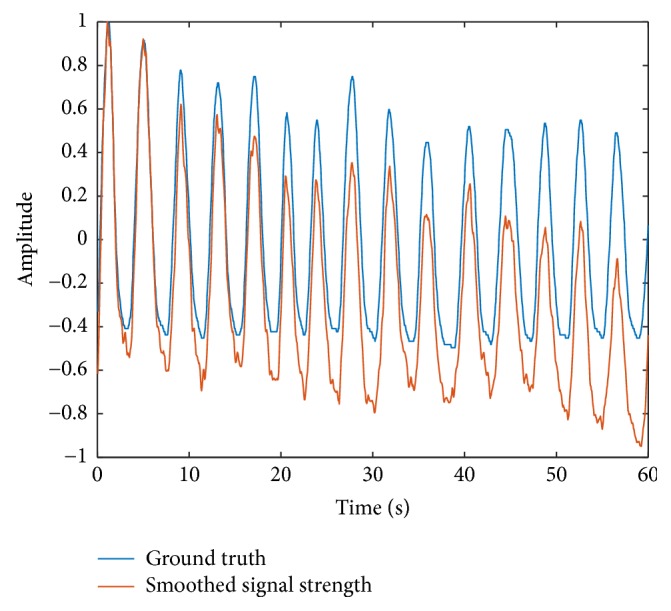
Signal strength fluctuation caused by the environment while testing.

With the estimated respiration waveform, we can derive the respiration rate of the tester. To simplify the algorithm, instead of frequency domain analysis, we want to use simple time domain analysis to detect the respiration rate by counting the number of peaks and valleys in the strength signal. To accurately count the peaks and valleys, a threshold is required, and by comparing the signal strength with the threshold, we can get the respiration period and respiration rate. However, in some cases, the signal strength may fluctuate severely because of the changing environment, just as Figure 3 shows. To solve this problem, we conduct dynamic threshold estimation at the third stage before calculating the respiration rate in the fourth stage. We calculate the dynamic threshold *P*_th_ by averaging *W*_th_ continuous points in ${\bar{P}}_{m}$.

(12) $$\begin{array}{c} P_{th}\left(\;m\;\right)=\frac{1}{W_{th}}\overset{\left(\left\lfloor m/W_{th}\right\rfloor +1\right)*W_{th}}{\underset{i=\left\lfloor m/W_{th}\right\rfloor *W_{th}+1}{\sum }}{\bar{P}}_{i},\;m=1,2,3,\ldots . \end{array}$$

The dynamic threshold *P*_th_ varies as the signal strength ${\bar{P}}_{m}$ changes.

**Figure 4:**
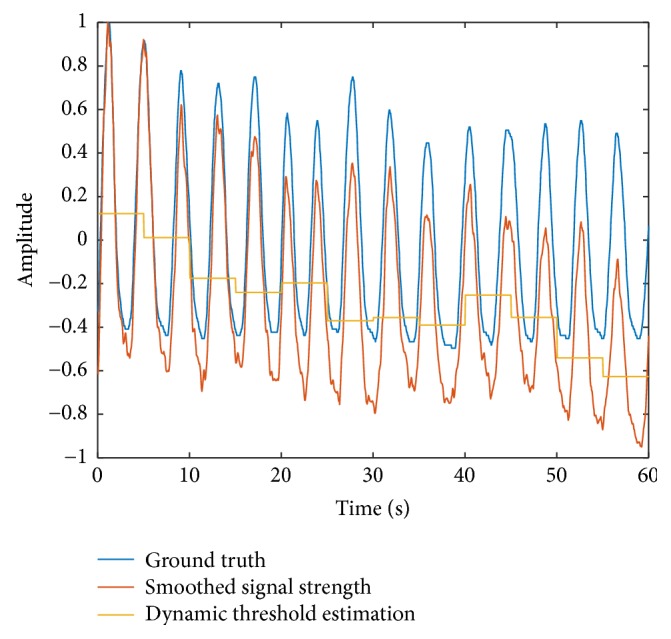
Dynamic threshold estimation.

**Figure 5:**
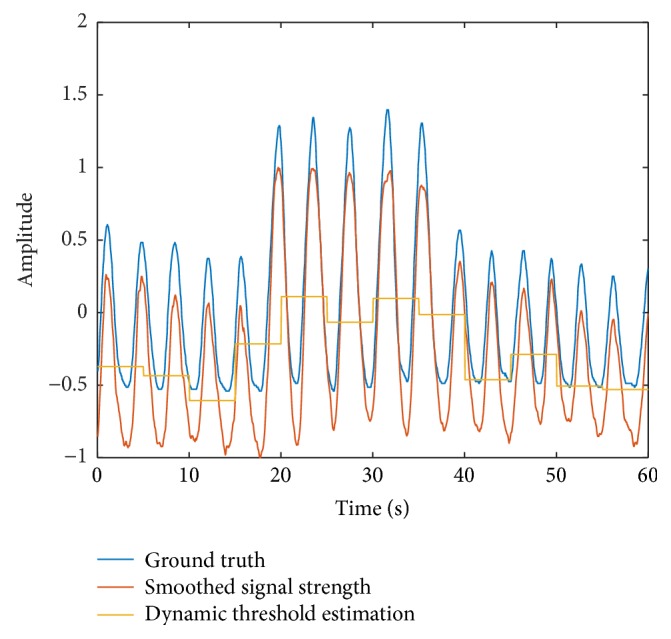
Dynamic threshold estimation under deep breath.

At the last stage, we use the smoothed signal strength ${\bar{P}}_{m}$ and the dynamic threshold *P*_th_ to estimate the respiration rate of the tester. We record the times that the estimated respiration waveform passes through the threshold, and then we get the period of the breathing. Using the average value of several recent periods, we can get the estimated respiration rate. Using this algorithm, we get Figure 4. This figure shows that our algorithm works well and gets a mean estimation error of 0.32 bpm in this example. We also test the case of deep breath; the algorithm still performs well as Figure 5 shows. The mean estimation error under deep breath is 0.35 bpm.

## 4. System Evaluation

### 4.1. System Implementation and Evaluation Setup

**Figure 6:**
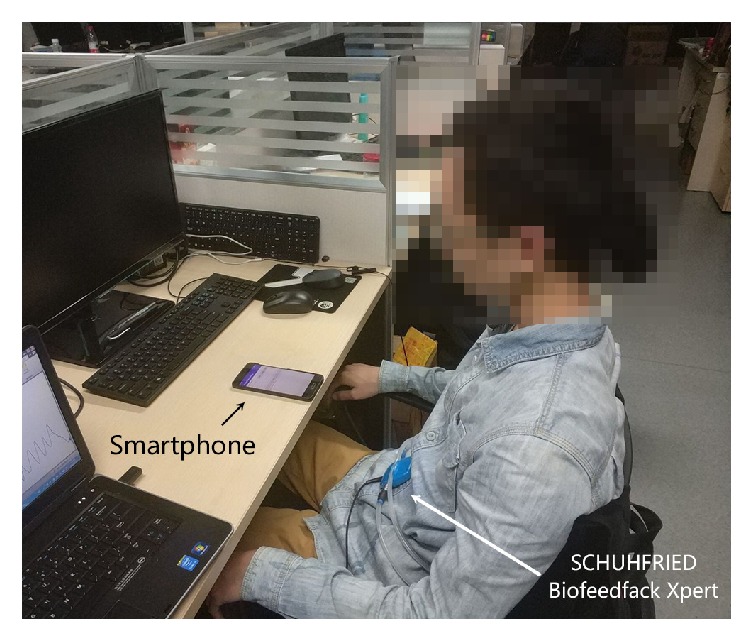
Experimental scenario in the office.

We develop an Android application to implement our algorithm on smartphones and evaluate the performance under various scenarios. We conduct experiments on two smartphones, a Xiaomi MI5 and a Samsung Galaxy S4, which are both based on the Android OS. Our application is developed with a minimum version of Android 4.0.4 and it works well on both smartphones. In our system, to generate ultrasound, we first generate a sound file in Pulse Code Modulation (PCM) format, and then we use AudioTrack in Android to play the generated sound file. The main speaker of the smartphone is used to transmit the ultrasound signal. In the receiving end, we use one microphone to receive the reflected signal, which works on the mono record mode instead of the stereo mode to reduce the complexity of the computation. The ultrasound signal is at the frequency of 20 kHz. The sampling rate of the microphone is 48 kHz which is the maximum sampling rate that most smartphones can support. To achieve the ground truth, we use the SCHUHFRIED Biofeedback Xpert [26] to monitor the respiration rate of the testers.  Figure 6 shows the experimental scenario in the office environment.

In the remaining part of this section, without specific instruction, the parameters are set as follows: sampling rate of the smartphone *F*_*s*_ = 48 kHz and coefficient* K* = 2400. Thus, the sampling rate of *P*_*m*_ is at a frequency of *F*_*p*_ = *F*_*s*_/*K* = 20 Hz. The window size of moving average filter *W* = 5. The window size of dynamic threshold estimation *W*_th_ = 100.

### 4.2. Evaluation Results

**Figure 7:**
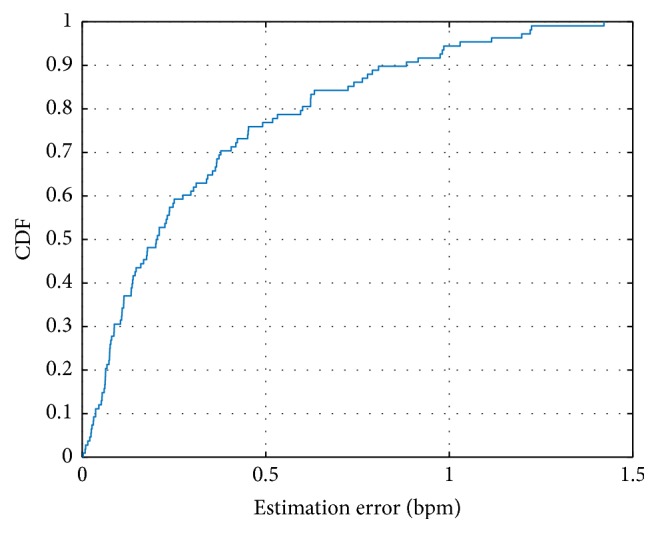
CDF of the estimation error.

We evaluate the overall performance of the system and calculate the cumulative distribution functions (CDF) of estimation errors on the respiration rate, when the distance between the smartphone and the tester is 15 cm. As Figure 7 shows, over 90% of the results have an estimation error under 0.8 bpm. The medium estimation error is 0.2101 bpm while the mean estimation error is 0.4137 bpm. Traditional medical respiration monitoring devices usually allow an error of 5%, considering that a normal breath rate is usually 15–20 bpm, and our estimation error is less than 4%, which is accurate enough for daily use.

**Figure 8:**
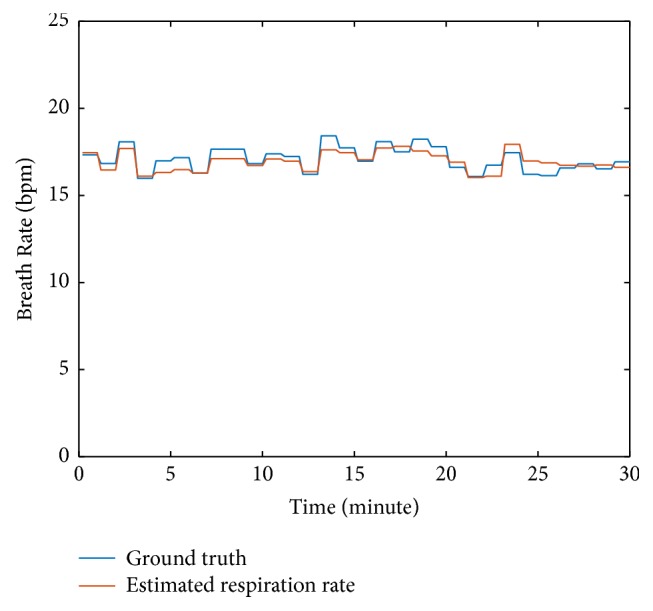
Respiration rate estimation result for 30-minute testing.

Figure 8 shows the respiration rate comparison for a test that lasts for 30 minutes. From this figure, we find that the estimated breathing rate follows the ground truth well. Due to the convenience of the smartphones, they are suitable for our algorithm to do a long-term monitoring.

Compared with Wang et al.'s work [21], we get similar results in estimation error with a much simpler algorithm. They employed sonar phase data to get the breath rate, resulting in high complexity in algorithm. In their work, the estimation error at a distance of 10 cm is about 0.3 bpm, which is very close to our results, but we achieve similar results using a much simpler algorithm.

### 4.3. Impact of Different Factors

**Figure 9:**
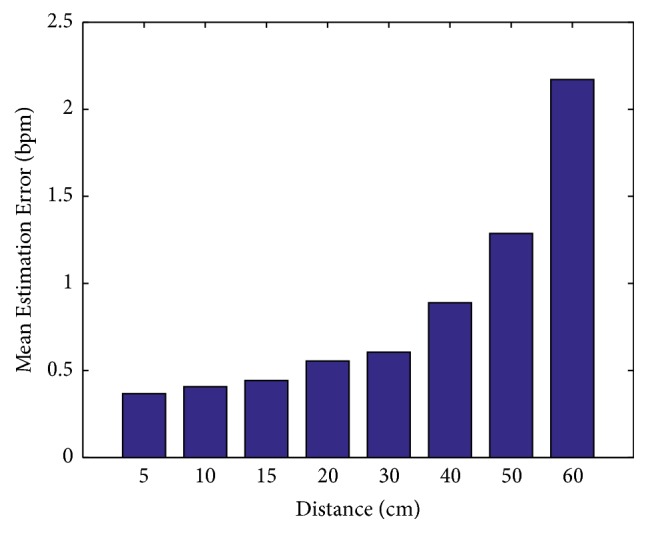
Estimation error versus the distance between the user and the smartphone.

*Impact of Distance.* Figure 9 shows the impact of distance between the microphone and people's chest. When the distance gets longer, the mean error gets larger. With the increase of distance, the signal attenuation becomes severe and the amount of reflected ultrasound signal becomes less. Thus, the energy change caused by the reflected ultrasound signal becomes more inconspicuous; therefore, the estimation accuracy will be reduced. We can see that our algorithm works well when the distance is under 40 cm. The mean estimation error is around 0.5 bpm under a distance of 30 cm and it is acceptable for daily use. When the distance is 40 cm, the mean estimation error is almost 1 bpm.

**Figure 10:**
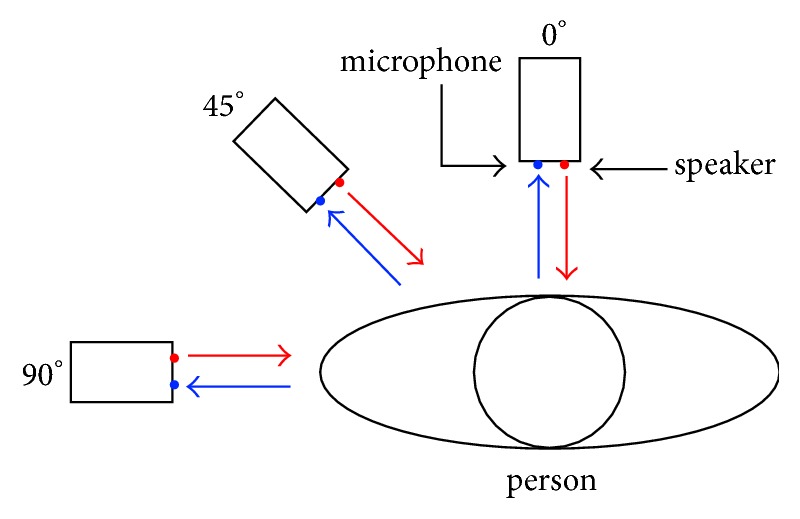
Orientation of the smartphone.

*Impact of Orientation.* Table 1 shows how the orientation influences the mean estimation error. In this experiment, we keep the distance at 10 cm and control the angle between the smartphone and the tester as Figure 10 shows. We consider three cases when the angle is equal to 0, 45, and 90 degrees, respectively. At 0 degrees, the person directly faces the smartphone, and we get the best performance at a mean estimation error of 0.39 bpm. At 45 degrees, the chest movements become less clear compared with 0 degrees. So, we get a worse mean estimation error at 0.50 bpm. At 90 degrees, the estimation error of 0.62 bpm is the worst due to the minimum chest movements in these three situations. Despite being much worse than the situation of facing the smartphone, the results at 90 degrees are still acceptable. The system can work in all orientations, because during breathing, the chest moves at both the front and side directions. However, the performance is optimal when the tester directly faces the smartphone.

*Results on Different Persons.* Table 2 shows whether the tester influences the estimation error a lot. In the experiment, we test three persons (two men and one woman) as shown in Table 3. The experiment is done at 10 cm distance in the office. We can see that there are small differences between different persons. These small differences may be caused by environmental noise, different chest movement length, and experimental error. The mean estimation errors of these three persons are all between 0.4 and 0.5 bpm, which is an acceptable result. Thus, our algorithm works well on different persons.

*Impact of Different Smartphones. *In this experiment, we use two smartphones, Xiaomi MI5 and Samsung Galaxy S4. From Table 4, we can see that the MI5 has a smaller mean estimation error than the S4. This is because the structures of the two smartphones are slightly different. For MI5, the speaker and microphone are both at the bottom of the phone. This means that when you put it on the desk, the speaker and microphone are directly pointing to the chest of the person. In contrast, the speaker of the S4 is on its back and its microphone is at the bottom. Thus, when we put the S4 on the desk, the volume may be reduced due to the position of the speaker. So, the received signal of MI5 is stronger than that of S4 and MI5 achieves a better performance in the experiment. We now know that the position of the speaker and the microphone does matter. A smartphone with its speaker and microphone at the bottom usually yields a better result. However, even when the speaker is at a relatively bad position, the performance is still acceptable.

*Impact of Various Testing Scenarios.* Table 5 shows the impact of different scenarios. We test four scenarios including office, dormitory, library, and office with music playing. They achieved mean estimation errors of 0.48, 0.43, 0.31, and 0.78 bpm, respectively. We get the minimum mean estimation error at the library, because in the library, it is quiet and there are a few objects around, which results in the minimum impact of the environment, and thus it achieves the minimum mean estimation error. In the dormitory and office, the situation is similar: crowded room with computer and air conditioners running, even some people talking with others. The noise generated from machines and people does influence the performance of our experiment. Because our algorithm is based on the energy of the signal, a loud noise may cover the signal that we want, resulting in bad performance. We further run experiments in the office with music playing to verify the impact of noise. The mean estimation error with music is 0.78 bpm, which is much larger than the general situation. This shows that a loud noise does make the performance worse. Furthermore, the music has a more severe impact than people's talking. That is because the frequency domain of music is relatively higher than people's voice; therefore, music has a higher influence on the 20 kHz signal that we use to monitor the breath. We did not test our system under the scenario when the tester is running, because all existing works [17, 21] conduct their experiments in a stable scenario. The experiments validate that, even in noisy scenarios, we can get mean estimation errors less than 1 bpm. In general scenarios, the mean estimation error is about 0.5 bpm, which is accurate enough for daily use.

**Figure 11:**
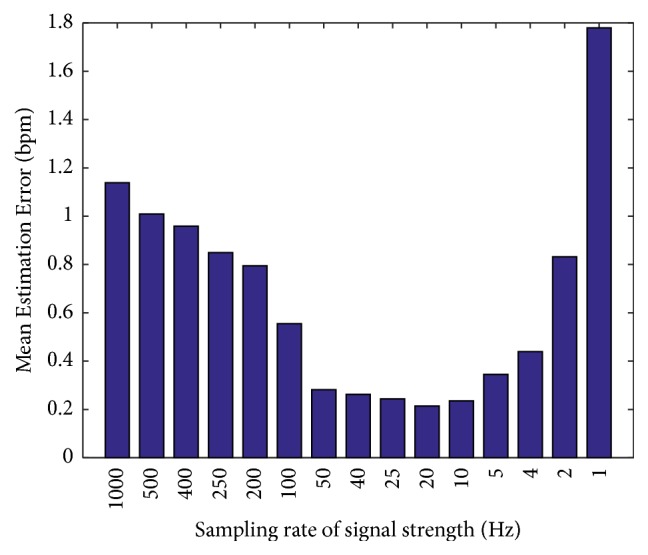
Mean estimation error versus sampling rate of signal strength *F*_*p*_.

**Figure 12:**
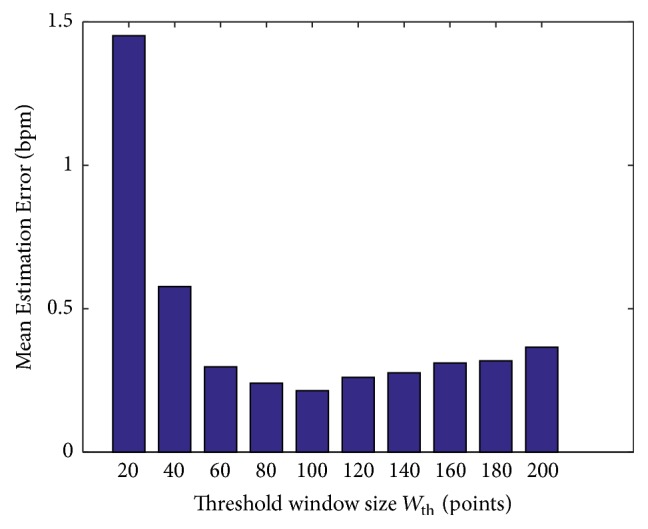
Mean estimation error versus threshold window size *W*_th_.

*Impact of Different Parameters. *Figures 11 and 12 show the reason why we choose the sample frequency of signal strength *F*_*p*_ equal to 20 Hz and the moving window size *W*_th_ equal to 100 points in our algorithm. We tried different parameters in our algorithm. For sampling rate *F*_*p*_, we get the minimum mean estimation error at 20 Hz. Although it is acceptable from 5 Hz to 50 Hz, we choose 20 Hz because the medical device SCHUHFRIED Biofeedback Xpert [26], which we use as our ground truth, also works at a sampling rate of 20 Hz. And for window size *W*_th_ of dynamic threshold estimation, we get the minimum estimation error at *W*_th_ = 100 points.

## 5. Conclusion

In this paper, we proposed a smartphone-based respiration rate detection system based on single-frequency ultrasound signals. The proposed system can track the movement of the human chest by observing the signal strength of the recorded ultrasound data. We implemented our system on an Android smartphone and conducted extensive experiments to show the feasibility and accuracy of our system. The results show that this system can achieve accurate respiration rate estimation under various scenarios.

## Figures and Tables

**Table 1:** Estimation error versus the orientation of the smartphone.

Orientation (degree)	Mean estimation error (bpm)
0	0.39
45	0.50
90	0.62

**Table 2:** Estimation errors on different persons.

Person number	Mean estimation error (bpm)
1	0.41
2	0.45
3	0.42

**Table 3:** Subjects information.

Person number	Gender	Age	Height (cm)	Weight (kg)
1	Male	23	172	65
2	Male	22	175	50
3	Female	22	160	50

**Table 4:** Estimation errors on different smartphones.

Phone	Mean estimation error (bpm)
Xiaomi MI5	0.37
Samsung Galaxy S4	0.41

**Table 5:** Estimation errors in different testing scenarios.

Scenarios	Mean estimation error (bpm)
Office	0.49
Dormitory	0.44
Library	0.31
Office (with music)	0.78
